# Proteomic analysis of cholera toxin adjuvant-stimulated human monocytes identifies Thrombospondin-1 and Integrin-β1 as strongly upregulated molecules involved in adjuvant activity

**DOI:** 10.1038/s41598-019-38726-0

**Published:** 2019-02-26

**Authors:** Manuela Terrinoni, Jan Holmgren, Michael Lebens, Maximilian Larena

**Affiliations:** 1 Department of Microbiology and Immunology and University of Gothenburg Vaccine Research Institute (GUVAX), Institute of Biomedicine, Sahlgrenska Academy at University of Gothenburg, Box 435, SE-405 30, Gothenburg, Sweden; 2 Department of Organismal Biology, Uppsala University, Norbyvägen 18C, SE-753 26, Uppsala, Sweden

## Abstract

Cholera Toxin (CT) as well as its related non-toxic mmCT and dmLT mutant proteins have been shown to be potent adjuvants for mucosally administered vaccines. Their adjuvant activity involves activation of cAMP/protein kinase A (PKA) signaling and inflammasome/IL-1β pathways in antigen presenting cells (APC). To get a further understanding of the signal transduction and downstream pathways activated in APCs by this group of adjuvants we have, employing quantitative proteomic analytic tools, investigated human monocytes at various time points after treatment with CT. We report the activation of three main biological pathways among upregulated proteins, peaking at 16 hours of CT treatment: cellular organization, metabolism, and immune response. Specifically, in the further analyzed immune response pathway we note a strong upregulation of thrombospondin 1 (THBS1) and integrin β1 (ITGB1) in response to CT as well as to mmCT and dmLT, mediated via cAMP/PKA and NFKB signaling. Importantly, inhibition *in vitro* of THSB1 and ITGB1 in monocytes or primary dendritic cells using siRNA abrogated the ability of the treated APCs to promote an adjuvant-stimulated Th17 cell response when co-cultured with peripheral blood lymphocytes indicating the involvement of these molecules in the adjuvant action on APCs by CT, mmCT and dmLT.

## Introduction

Cholera toxin (CT) has for a long time been of great interest in mucosal immunology due to its strong adjuvant properties^1^. Mucosal administration of CT with an antigen substantially increases host mucosal as well as systemic humoral and cellular immune responses, including mucosal IgA and serum IgG and IgA antibody responses and cellular CD4^+^ and CD8^+^ T cell responses^2,3^. The molecular mechanisms by which CT works as a potent enterotoxin in the pathogenesis of cholera have been clarified in considerable detail (see [6] for a recent review): CT binds to GM1 ganglioside receptors on gut epithelial cells via its B subunit pentamer (CTB) leading to cellular uptake of the toxin and release from the endoplasmic reticulum of its toxic-active A subunit (CTA), which latter by ADP-ribosylating the α subunit of the GTP-binding regulatory protein G*s* induces adenylate cyclase activation, resulting in elevated cAMP levels. In the intestine, cAMP serves as a second messenger that induces protein kinase A (PKA)-dependent chloride channel activation resulting in massive fluid secretion and hence clinically presenting as often life-threatening watery diarrhea.

The strong enterotoxicity of CT, as well as of its heat-labile toxin (LT) analogue in enterotoxigenic *E. coli*, precludes the use of the native toxins as adjuvants for orally co-administered vaccines. It is therefore important to develop clinically safe mucosal adjuvants. Thus, non-toxic derivatives of CT and LT with largely retained adjuvant activity, the double-mutant LT (dmLT) and the multiple-mutated CT (mmCT), were recently generated^4–6^.

The adjuvant mechanisms of CT or LT remain less well defined than the enterotoxic mechanisms. Both toxins have been found to affect the function of many types of immunologic cells, including various types of APCs and different types of lymphocytes including B cells, different subsets of T helper and effector cells as well as regulatory T cells^7–13^. Substantial evidence indicates that the primary action is on dendritic cells (DCs) and other APCs in ways promoting their T cell activating function^10,11^. In human and murine DCs as well as other APCs such as monocytes, the activation of the cAMP/PKA pathway and activation of inflammasome-dependent IL1 signaling, have been found to be central in the adjuvant function of CT leading to enhanced activation of helper T cells, predominantly Th17 but also Th1 and Th2 cells. Notably, mmCT and dmLT were also found to use similar pathways as CT in stimulating antigen presentation and T cell activation in APCs despite inducing approximately 1000-fold lesser levels of cAMP intracellularly; apparently, the minimal cAMP induced by mmCT and dmLT is both sufficient and necessary for the adjuvant action^10^. Associated with these effects we have also found an important role of S100 calcium binding protein A4 (S100A4) and calcium signaling in the adjuvant function induced by CT as well as mmCT on DCs and other APCs^11^.

However, our knowledge of molecular signal transduction mechanisms induced by CT in APCs remains incomplete. To gain further insights about immunomodulatory proteins and signaling pathways affected by CT in APCs, we have here carried out high-throughput proteomic analysis of human monocytes after treatment for various times with CT. We show that CT significantly altered global protein expression in monocytes, with peak responses observed after 16 h of treatment. The three most prominent key biological processes that were induced include cellular organization, metabolism and immune response. Among the CT-induced immune response proteins, the most strongly upregulated ones were the immunomodulatory molecules thrombospondin 1 (THSB1) and integrin β1 (ITGB1), whose increased expression was also confirmed by ELISA and flow cytometry, respectively. Experiments showed that the specific inhibition of either THSB1 or ITGB1 in monocytes or DCs resulted in significant dampening of the capacity of these APCs to promote increased IL-17 production by co-cultured peripheral blood lymphocytes, indicating the functional importance of THSB1 and ITGB1 in the adjuvant action by CT. Further, the blocking of cAMP production or the inhibition of PKA activity or NFKB signaling in APCs were found to abrogate the increased expression of THSB1 and ITGB1 along with the Th17 promoting activity induced by either of CT, mmCT or dmLT supporting that all of these effects depend on adjuvant-induced cAMP/PKA signaling.

## Materials and Methods

### Cells

Fresh PBMCs from buffy coats from healthy human blood donors were purified by density-gradient centrifugation using Ficoll-Paque (GE Healthcare Bio-sciences). CD4^+^ T cells were obtained by negative selection and CD14^+^ monocytes were isolated by positive selection, in each case using magnetic beads in accordance with the manufacturer’s protocol (Miltenyi Biotec). Dendritic cells were purified with the Blood Dendritic Cell Isolation Kit II from Miltenyi Biotec in a two-step procedure also using magnetic beads. Cells were maintained at 37 °C with 5% CO_2_, in DMEM-F12 complete medium (Life Technologies) supplemented with 1% gentamicin (Sigma-Aldrich; 50 mg/ml) and 5% human AB+ serum (Sahlgrenska University Hospital blood bank).

### Trypan blue staining

CD14^+^ monocytes samples were incubated in triplicates for 0, 2 h and 16 h without stimulus (NS) and for 16 h at 37 °C with 5% CO_2_ with either 1 µg/mL CT, or 1 µg/mL CT plus 20 µM protein kinase A inhibitor (H-89), or 1 µg/ml CT plus 20 µM NFKB inhibitor (CAPE). The inhibitors were added 1 h prior to treatment with CT. Cell viability was measured by trypan blue staining counting the living cells. This was done by carefully suspending the incubated cells by repeat pipetting, mixing the suspended cells in triplicates with trypan blue buffer (dilutions 1:2 and 1:5) and counting the live (non-stained) and dead (blue-stained) cells using a Bürker chamber under a microscope. The mean and standard error of the mean (SEM) live cells numbers were calculated.

### Cell preparation for Proteomics

Both for the purpose of getting enough cells for the proteomic analysis and to minimize the influence of individual variations, purified monocytes from the buffy coats of 18 healthy human blood donors were treated with 1 µg/ml CT for 2 h, 4 h, 6 h, and 16 h or incubated untreated for 2 h [non-stimulated (NS) controls] at 37 °C with 5% CO_2_. After thorough washing 3 times with PBS, cells were snap-frozen in liquid nitrogen and stored at −70 °C. Cells were transported in dry ice to Proteomics & Mass Spectrometry Facility, Donald Danforth Plant Science Center for proteomic analysis. Cells were pooled such that each replicate is a pool of 6 individuals, yielding a total 15 samples for analysis (triplicates of 4 CT treatment time-points samples and 1 NS sample). We verified that non-stimulated cell counts did not change between the different time points and also did not change in CT-stimulated cells after 16 h. In addition, trypan blue positive cells were consistently below 1%, indicating negligible cell death at all time points and treatments (Table S3- Supplemental File 1).

For protein analyses, the cell samples were lysed in a denaturing lysis buffer [100 mM Tris-HCl pH 8.0, 8 M urea, 1 mM ethylenediaminetetraacetic acid (EDTA), 1 mM ethylene glycol-bis(β-aminoethyl ether)-*N*,*N*,*N*′,*N*′-tetraacetic acid (EGTA), 1 mM tris(2-carboxyethyl)phosphine (TCEP), 1X PhosStop inhibitor cocktail (Roche), 1X phosphatase inhibitor cocktail II (EMD Millipore)] and sonicated on ice at 50% power with four one-sec pulses. The protein lysates of the total 15 samples were determined by a protein assay kit (Cytoskeleton). In addition, a reference protein pool, generated by combining equal amounts of all samples tested, were prepared. Finally, 100 µg of all test samples and the reference pool were precipitated using the 2-D Clean-Up kit (GE Healthcare) according to the kit instructions, and the precipitated pellets were stored at −80 °C.

### Peptide Digestion and TMT 10plex Labeling

For isobaric labeling, the precipitated protein pellets were dissolved in 40 µL of 100 mM Tris-HCl, pH 8.0. Solubilized proteins were reduced with the addition of 2.1 µL of 100 mM TCEP (5 mM final) with shaking in a thermomixer (1000 RPM) for 30 min at room temperature, and then alkylated by the addition of 3.4 µL of 550 mM iodoacetamide (40 mM final) with shaking in a thermomixer (1000 RPM) for 30 min at room temperature in the dark. The alkylation reaction was quenched by addition of 4.5 µL of 500 mM 1,4-dithiothreitol (DTT-40 mM final) with shaking in a thermomixer (1000 RPM) for 15 min at room temperature. Proteins were acetone precipitated according to the TMT (Tandem Mass Tag™) 10plex reagent kit (Thermo Scientific) instructions and precipitates dissolved with 100 µL of 100 mM triethylammonium bicarbonate (TEAB) before endoprotease digestion with 2 µg of a Trypsin/Lys-C mixture (Promega) corresponding to a 50:1 protein:protease ratio (w/w) and incubation with shaking (1000 RPM) in a thermomixer overnight at 37 °C. The peptide concentration was assessed and 50 µg of digested peptides from each sample were labeled according to the TMT 10plex reagent kit instructions with the labeling scheme detailed in Table S1 (Supplemental File 1). Briefly, TMT reagents were brought to room temperature and dissolved in anhydrous acetonitrile. Peptides were labeled by the addition of each label to its respective digested sample. Labeling reactions were incubated without shaking for 1 h at room temperature. Reactions were terminated by the addition of hydroxylamine. The labeled digests for each experiment were pooled and dried by evaporation.

### High pH Reverse Phase Fractionation

The dried peptide mixture was dissolved in 55 μL of mobile phase A (10 mM ammonium hydroxide). 50 μL of the sample was injected onto a 2.1 × 150 mm XSelect CSH C18 column (Waters) equilibrated with 3% mobile phase B (10 mM ammonium hydroxide, 90% Acetonotrile). Peptides were separated using a similar gradient as previously described^14^ with the following gradient parameters (Table S2-Supplemental File 1) at a flow rate of 0.2 mL/min. 60 peptide fractions were collected corresponding to 2.5 min each. Ten pooled samples were generated by concatenation^15^ in which every 10th fraction was combined (ie: 1, 11, 21, 31, 41, 51; next 2, 12, 22, 32, 42, 52, etc., six pools total).

### LC-MS Analysis

Each pooled sample (~10 µg) was reconstituted by vortexing in 50 µL of 5% acetonitrile/0.1% formic acid. Samples were transferred to autosampler vials for LC-MS analysis, and 5 µL of each sample was analyzed by high resolution HCD MS/MS with a Dionex RSLCnano HPLC coupled to a QExactive (Thermo Scientific) mass spectrometer using a 2 h gradient. Peptides were resolved using a 75 µm × 25 cm PepMap C18 column (Thermo Scientific). MS1 resolution was set to 70,000 and MS/MS resolution was set to 35,000.

### Protein Identification

All MS/MS samples were analyzed using Proteome Discoverer 2.1 (Thermo Scientific). Proteome Discover was set up to search the NCBInr *Homo sapiens* database (GI TaxID = 9606, v2015-12-05) assuming the digestion enzyme trypsin. The HCD spectra MS/MS spectra were searched with a fragment ion mass tolerance of 0.02 Da and a parent ion tolerance of 10 ppm. Oxidation of methionine was specified as a variable modification, while carbamidomethyl of cysteine and TMT labeling was designated at lysine residues or peptide N-termini were specified in Proteome Discoverer as static modifications. MS/MS based peptide and protein identifications and quantification was also performed in Proteome Discover 2.1. A 1% FDR threshold was set for peptide identifications. Proteins that contained similar peptides and could not be differentiated based on MS/MS analysis alone were grouped. Normalized and scaled protein/peptide abundance ratios were calculated using the mean abundance of the three replicates of a condition over the abundance value of the reference pool (131TM).

### Dataset

The mass spectrometry proteomics data have been deposited to the ProteomeXchange Consortium (http://proteomecentral.proteomexchange.org) via the PRIDE partner repository^16^ with the dataset identifier <px-submission #287019>.

### Bioinformatic Analysis

Since comparisons were to be performed between data in the two different labeling sets, the protein expression was normalized to the pool as described by the equation “Exp_sample_normalized = Exp_sample / Exp_pool”^17^. Two-group comparisons were performed between NS vs CT2h, NS vs CT4h, NS vs CT6h, and NS vs CT16h. A two-tailed unpaired student t-test was performed for each comparison and proteins with a p-value < 0.05 were considered “significantly altered”. A criterion of ${\rm{\ge }}$1.3 fold change was applied to further signify the up-regulated or down-regulated proteins^18,19^, which proteins are referred to as “significantly upregulated” or “significantly downregulated”. Significantly altered proteins that fulfilled the 1.3 fold change criterion in the different comparisons were analyzed for the function enrichment using the online tool David Bioinformatics^20,21^ and Panther^22^ (http://pantherdb.org.) Functional Annotation Tool via DAVID’s Bioinformatic Online Resource was run using default parameters as recommended^20^. All pathways listed in Chart Report had to pass the set thresholds (by default, Ease Score of <= 0.1 and Minimum Count >= 2) to ensure that only statistically significant ones were displayed. All pathways listed in our analysis have Fisher Exact test p values < 0.05.

### Cell stimulations and co-culture model

Inhibition of PKA signaling was done with addition of H-89 (Sigma-Aldrich; 20 µM) and inhibition of NFKB was performed with CAPE (Sigma-Aldrich; 20 µM) with cells then incubated with the inhibitors for 1 h prior to the subsequent treatment with adjuvants. Inhibition of the guanine nucleotide-binding protein G subunit alpha (GNAS), THSB1, ITGB1, the arachidonate 5-lipoxygenase (ALOX-5), or nonspecific ALL-STAR negative control by siRNA-induced gene silencing were performed following manufacturer’s instructions (Qiagen). Briefly, siRNA complexes were prepared with individual siRNAs incubated with Transfection Reagent (Qiagen) and serum free media for 10 minutes at 25 °C, and added to pre-seeded CD14^+^ monocytes or DCs (final concentration of 25 nM) for 20–24 h transfection at 37 °C with 5% CO_2_. Following washing of cells 3x with PBS, purified CD14^+^ monocytes (5 × 10^4^ per well) or DCs (1 × 10^4^ per well) were left untreated or stimulated with 1 µg/ml CT, mmCT, or dmLT, or with 1 mM cAMP analogue (dcAMP; N6,2′-O-dibutyryladenosine3′5′-cyclic monophosphate sodium salt; Sigma-Aldrich) for 16 h in 96-well round bottom plates. After adjuvant treatments with or without inhibitions, cells were washed with PBS 3x, and were co-cultured with autologous CD4^+^ T cells (5 × 10^4^ for monocytes per well or 1 × 10^5^ for DCs per well) with or without the addition of 10 ng/ml of a polyclonal stimulus of staphylococcal enterotoxin B (SEB, Sigma-Aldrich). After 3 days, culture supernatants were collected for IL-17A cytokine levels detection by ELISA.

### ELISA

Supernatants were stored at −70 °C prior to analysis. Levels of IL-17A or THSB1 were determined using ELISA following manufacturer instructions (R&D Systems). Assays were read at 450 nm within 30 minutes after last step of the procedure (BioTek).

### Flow Cytometry

PBMCs (1.5 × 10^6^/1.5 ml) were left untreated, or treated for 1 hr with H-89 or treated overnight with specific siRNA as described above, and then stimulated for 16 h with CT, mmCT, dmLT, or dcAMP. Cells were washed, and surface-stained with anti-CD19 Alexa700, anti-CD14 PeCy7, anti-HLA-DR APC-Cy7, anti-CD11c BB515-FITC, anti-CD4 A700, and anti-ITGB1 PE. After treatment with fluorochromes, cells were washed, fixed in 2% paraformaldehyde, and analyzed with LSRII Flow Cytometer (BD Biosciences) equipped with blue and red lasers. Results were analyzed and plotted with FlowJo software (Tree Star).

## Results

### Treatment of human monocytes with CT increases the expression of many proteins in a time-dependent manner

**Figure 1:**
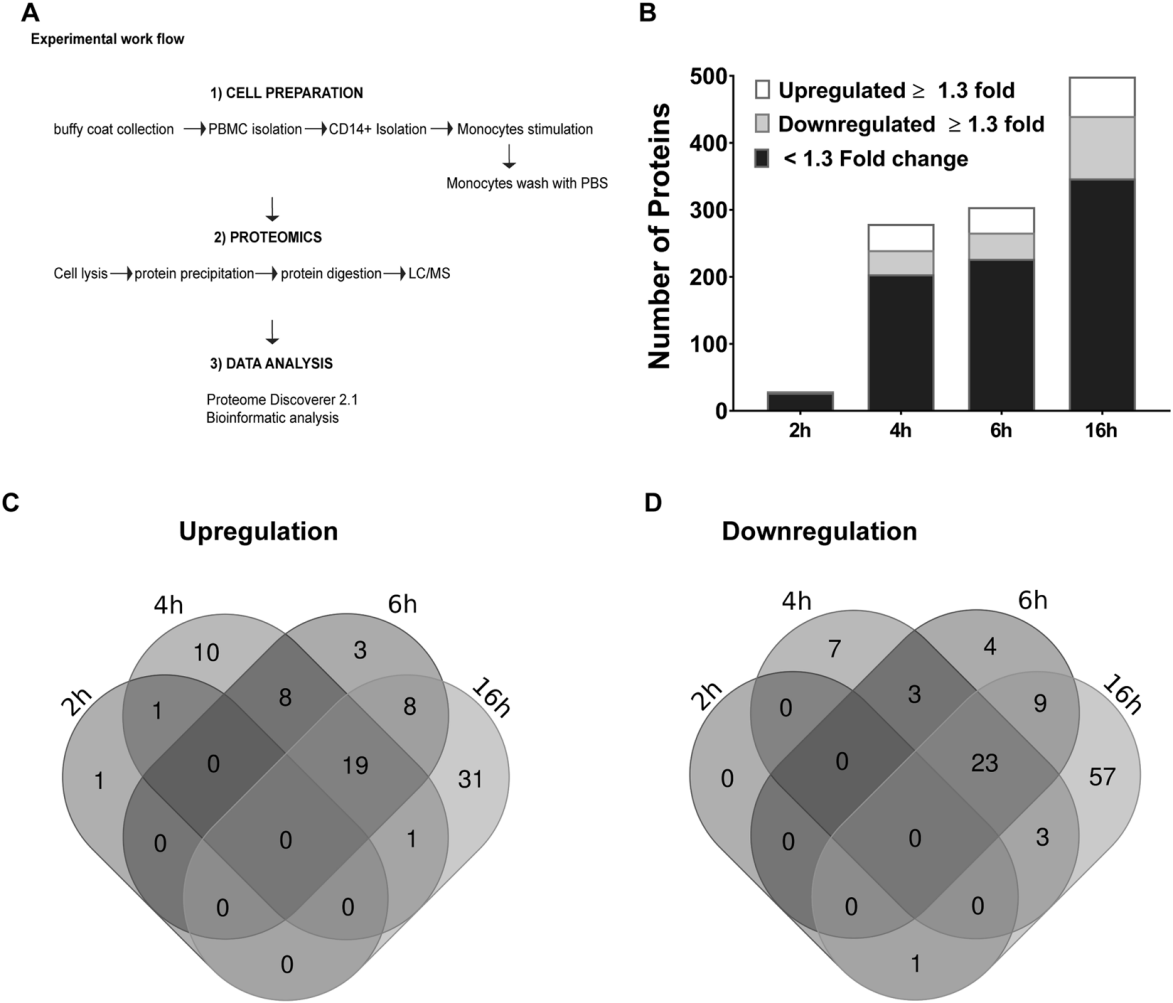
Global protein expression after CT-stimulation of human monocytes. Purified CD14^+^ monocytes were left untreated or treated with CT (1 µg/ml) for 2 h, 4 h, 6 h, or 16 h, washed, and processed for proteomic analysis (**A**). Bar graph (**B**) represents the numbers of proteins being significantly upregulated $\ge$1.3 fold (white shade), downregulated $\ge$1.3 fold (gray shade), or altered less than 1.3 fold (black shade) in CT-treated samples compared to untreated cells incubated for 2 h; control studies showed that there was no change in either treated or untreated cell numbers, even after 16 h incubation. Venn diagrams represent numbers of significantly upregulated (**C**) or downregulated (**D**) proteins at the different CT treatment time points

To examine the effect of CT on global protein expression in human monocytes, pooled protein extracts of monocytes from altogether 18 individuals treated with CT for 2, 4, 6 and 16 h or incubated in medium only for 2 h (NS), in three pools representing proteins from 6 individuals in each (altogether 15 samples), were analyzed by LC/MS (Fig. 1A); control studies demonstrated that NS cell numbers and cell viability did not change throughout the 16 hour incubation period (Table S3 - Supplemental File 1). The proteomic analysis revealed a total number of 4756 distinct proteins quantified, with 1731 similar proteins identified in all 15 samples. Two-group comparisons on normalized protein expression were performed between CT-treated vs NS samples, with a p-value of <0.05 considered as “significantly altered” and a 1.3-fold change up or down used as an additional cut-off criterion defining “significantly upregulated” or “significantly downregulated” proteins. The number of significantly altered proteins increased with the length of CT treatment, with 37, 204, 227, and 347 proteins being increased after 2, 4, 6, and 16 h of treatment with CT, respectively (Fig. 1B & Supplemental Tables 1–4). Likewise, the number of significantly upregulated or downregulated proteins also increased over time, with 2, 39, 38, 59 upregulated proteins and 1, 36, 39, 93 downregulated proteins after 2, 4, 6, and 16 h of CT treatment, respectively. Furthermore, a temporal increase in the number of significantly upregulated or downregulated proteins shared between time points was also observed; 8 significantly upregulated and 1 significantly downregulated proteins were found after CT treatment for either 4 or 6 h, and 19 significantly upregulated and 17 significantly downregulated proteins were found after treatment for both 6 and 16 h (Fig. 1C,D).

Consistent with these observations, hierarchal clustering analysis revealed a progressive temporal increase in global protein expression presenting in three distinct clusters: low level expression in NS and 2 h CT- treated samples, moderately increased protein expression in samples treated with CT for 4 or 6 h, and maximally increased protein expression in samples from monocytes treated with CT for 16 h (Figure S1 - Supplemental File 1). Taken together, these data show that CT treatment of human monocytes results in significant changes in global protein expression, with the most marked effects found after 16 h of CT treatment.

### Functional Enrichment Analysis identifies CT-induced metabolic and immune-related pathways

**Figure 2:**
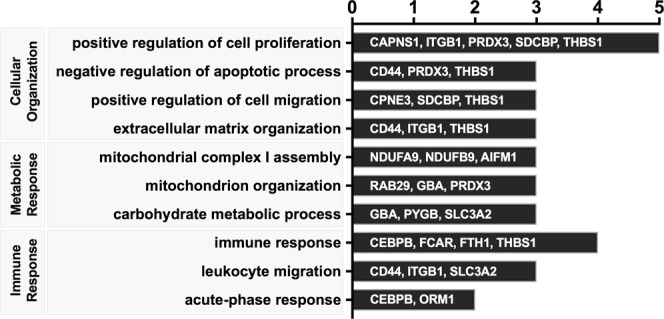
Functional enrichment analysis of upregulated proteins in CT-treated monocytes. The list of significantly upregulated proteins in monocytes treated with CT for 16 h were analyzed for functional enrichment using Gene Ontology Biological Process category of DAVID functional annotation tool. Bars with text show upregulated proteins in the three main biological pathway identified.

To identify relevant pathways induced by CT on human monocytes, functional enrichment analysis of annotated proteins was implemented. Given that the most significant change in protein expression was observed at 16 h of treatment with CT, we focused the analysis on CT-induced changes at this time point. Using Gene Ontology Biological Process category of DAVID functional annotation tool, the top ten pathways that were enriched from the list of significantly upregulated proteins cover three relevant functional processes: cell organization, metabolism and immune response (Fig. 2).

The cell organizational processes (positive regulation of cell proliferation and migration, extracellular matrix and mitochondrion organization, and negative regulation of the apoptosis) and metabolic processes (carbohydrate metabolism and mitochondrial respiratory chain I complex) signify a biologically active and thriving cell that reflects an immunologically functional APC^23^. This is further supported by enrichment of immune-related functional categories in CT-stimulated human monocytes such as immune response, leukocyte migration, and acute-phase response. Furthermore, similar biological processes were enriched when the list of significantly upregulated proteins were analyzed using PANTHER classification system (Figure S2-Supplemental File 1).

**Figure 3:**
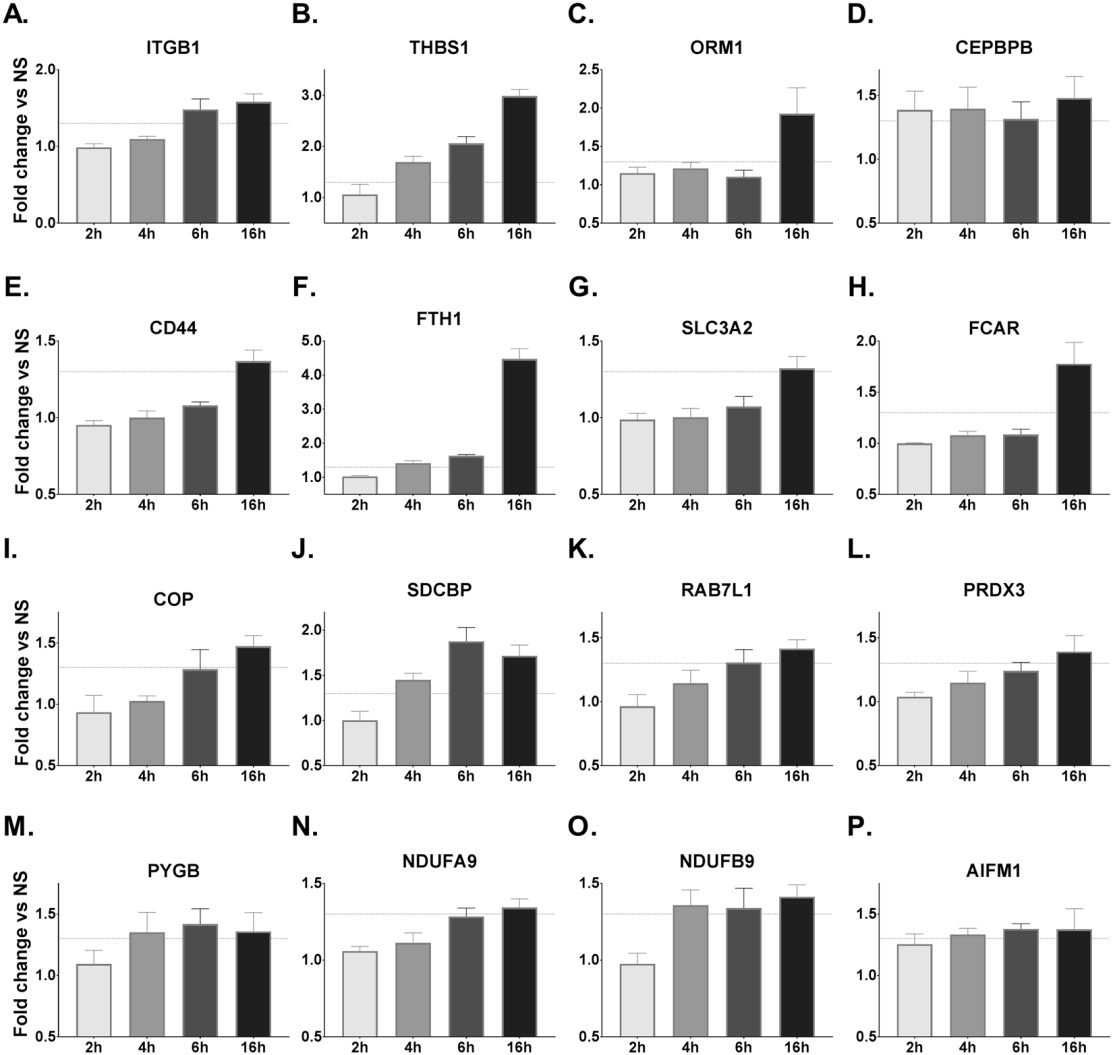
Kinetics of expression of relevant proteins in CT-stimulated monocytes. List of proteins of known relevance to biological processes were selected from the significantly upregulated proteins identified in Fig. 2. Bars represent means with SEM of protein expression relative to unstimulated cells and the dotted lines shows the 1.3-fold change cut off level for CT-treated vs non-stimulated samples (NS).

The kinetics of 16 individual upregulated proteins relevant to the aforementioned three major functional categories revealed a general trend of peak expression at later time points, mostly at 16 h (Fig. 3). Among these, 8 proteins were classified as immune-related with THBS1 being the strongest upregulated displaying an incremental increase in expression from 1.7-fold at 4 h, 2-fold at 6 h, and 3-fold at 16 h. To identify relevant protein-protein interactions amongst the proteins upregulated at 16 h, especially in relation to THBS1, we analyzed the dataset using the STRING database. Interestingly, we noted a close association between two immune related proteins, THSB1 and the beta-integrin receptor protein ITGB1 (Figure S3 - Supplemental File 1). For this reason, we focused our further analysis on whether or not THSB1 and ITGB1 play a role in the adjuvant action of CT.

### Inhibition of THSB1 and ITG1B expression in APCs abrogates the Th17 promoting adjuvant effect of CT

**Figure 4:**
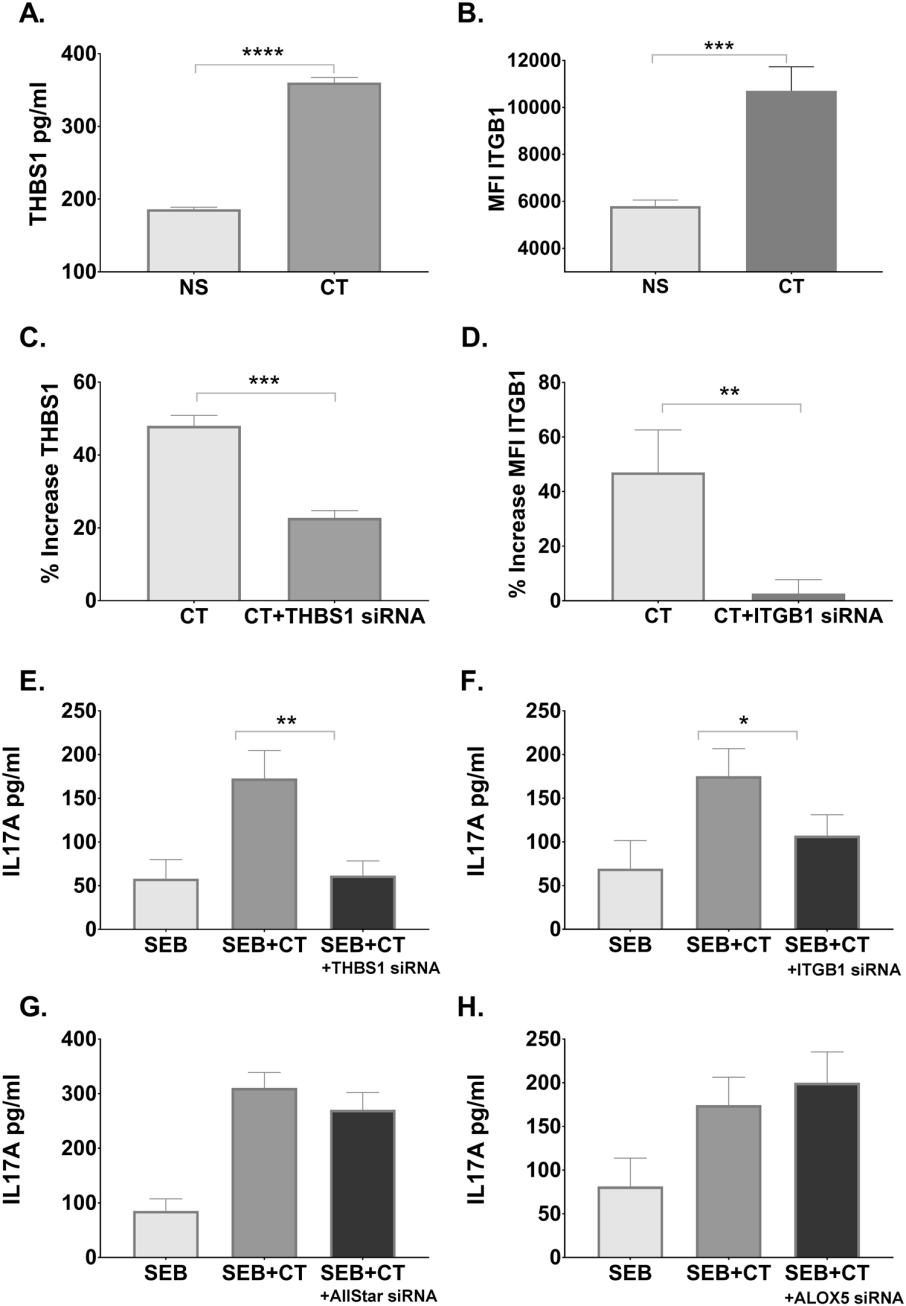
THBS1 and ITGB1 are required in APCs for promotion of Th17 responses by CT. THSB1 (**A**) and ITGB1 (**B**) protein expression were measured by ELISA and flow cytometry, respectively, in monocytes incubated with CT or left untreated for 16 h. Bars represent mean and SEM of THBS1 protein concentration in cell supernatants (**A**) or cell surface median fluorescence intensity (MFI) of ITGB1 (**B**). In C and D are shown that the increase in THSB1 (**C**) and ITGB1 (**D**) expression after CT treatment was significantly reduced after inhibition of the THSB1 (**C**) and ITGB1 (**D**) genes with specific siRNAs. (**E**–**H**) Purified CD14^+^ monocytes treated for 24 h with siRNAs against THBS1 (**E**), ITGB1 (**F**), All-Star Control (**G**), and ALOX5 (**H**), and subsequently incubated for 16 h with CT, were co-cultured with autologous CD4^+^ T cells plus the polyclonal stimulus SEB for 3 days whereafter IL-17A cytokine levels were measured in supernatants by ELISA. Bars show mean and SEM of IL-17A cytokine levels.

To independently confirm the ability of CT to induce expression of immune-related proteins THSB1 and ITGB1, we performed ELISA for determination of THSB1 levels and flow cytometric analysis for ITGB1 detection. As expected, exposure of monocytes with CT resulted in increased expression of both THSB1 and ITGB1 (Fig. 4A,B). We then tested whether THSB1 and ITGB1 are required for the adjuvant action of CT in a previously established monocyte-CD4^+^ T cell *in vitro* co-culture model assessment of adjuvanticity using specific siRNA inhibitors. After transfection with specific and control siRNAs, monocytes were stimulated with CT for 16 h. Following extensive washes, CT-treated and untreated monocytes were then co-cultured with autologous CD4^+^ T cells with or without SEB polyclonal antigen, and after 3 days IL-17A levels in the co-culture supernatants were measured by ELISA. First, we validated that overnight incubation of monocytes with THSB1 or ITGB1-specific siRNA resulted in significant reduction of the respective target proteins (Fig. 4C,D). As expected, human monocytes treated with control siRNA and CT showed significant enhancement of IL-17A responses. In contrast, co-culture of THSB1 or ITGB1-specific siRNA vs All-Star siRNA (Fig. 4G) control-treated monocytes with autologous CD4^+^ T cells resulted in the abrogation of CT-mediated enhancement of IL-17A responses (Fig. 4E,F). The abrogation of IL-17A responses was not observed in another independent control, when we inhibited ALOX5 (a potent pro-inflammatory mediator that was also significantly enhanced at 16 h based on proteomic analysis) with ALOX5-specific siRNA in monocytes (Fig. 4H).

### The induction of THSB1 and ITGB1 is dependent on cAMP-PKA pathway and on NFKB signaling

**Figure 5:**
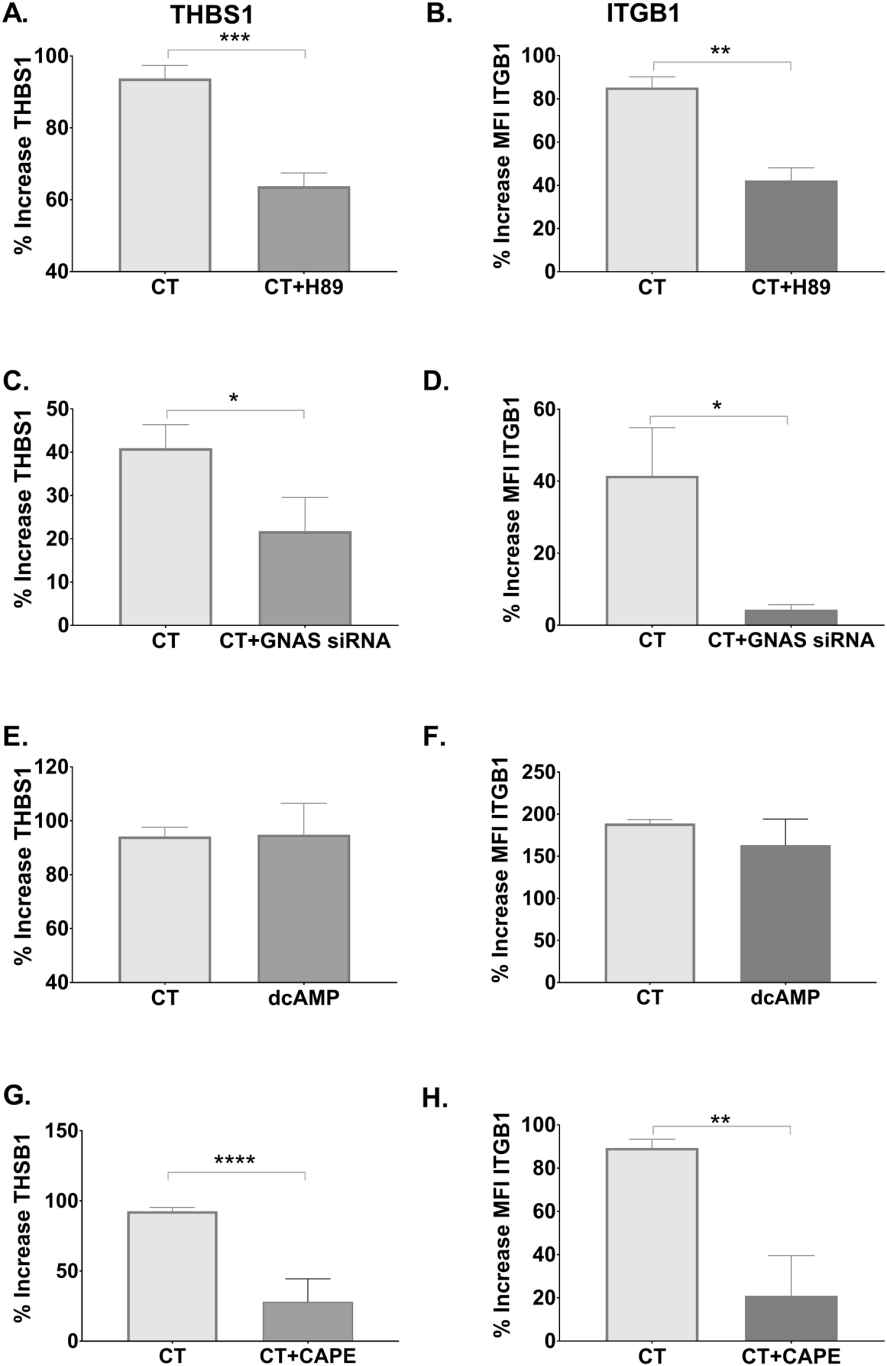
Promotion of TH17 responses via THSB1 and ITGB1 is cAMP/PKA-dependent. Purified CD14^+^ monocytes were left untreated or treated for 16 h with CT with and without the PKA inhibitor H-89 (**A**,**B**) or with or without the NFKB inhibitor CAPE (**G**,**H**) or with the cAMP analogue dcAMP (**E**,**F**). Expression of THBS1 (**A**,**E**,**G**) in cell supernatants was determined by ELISA and of ITGB1 (**B,F,H**) on the cell surface by flow cytometry. CD14^+^ monocytes were also left untreated or treated for 24 h with GNAS (Gsα)-specific siRNA, washed, and subsequently treated for 16 h with CT. Percentage differences in expression of THSB1 (**C**) or ITGB1 (**D**) in comparison with untreated cells are shown.

CT and the related mucosal nontoxic adjuvants were previously shown to mediate their Th17-promoting adjuvant effect in human APCs via cAMP/PKA signaling^10^ and via the canonical NFKB pathway (Terrinoni M. *et al*., manuscript in preparation). We tested the role of cAMP-PKA signaling also for the induction of THSB1 and ITGB1 with the use of a competitive inhibitor of cAMP-dependent PKA, H-89. Addition of H-89 to CT-treated monocytes resulted in reduced expression of THSB1 or ITGB1 (Fig. 5A,B). We further confirmed this when we treated monocytes with siRNA specific for Guanine Nucleotide-Binding Protein G Subunit Alpha (GNAS), a key protein that activates that cAMP-inducing adenylate cyclase enzyme. Treatment of monocytes with GNAS-specific siRNA resulted in reduced capacity of CT to induce expression of THSB1 or ITGB1 (Fig. 5C,D). Moreover, treatment of monocytes with an analogue of cAMP (dcAMP) resulted in enhanced expression of THSB1 and ITGB1 to levels that are comparable to those induced by CT (Fig. 5E,F). Lastly, to test the dependence of NFKB pathway from the expression of THSB1 and ITGB1, we used a specific NFKB inhibitor, CAPE. Addition of CAPE to CT-treated monocytes resulted also in a reduced expression of THSB1 or ITGB1 (Figure G,H). Vitality trypan blue testing and cell counting confirmed that none of the protein inhibitors (H-89 and CAPE) had any detectable toxic effects on monocytes at the concentrations used in the experiments (Table S3 - Supplemental File 1).

Altogether, these data demonstrate that the CT-induced increased expression of THSB1 and ITGB1 is dependent on the linked cAMP-PKA-NFKB signaling.

### THSB1 and ITGB1-dependent adjuvant effect of CT is also observed in human primary dendritic cells

**Figure 6:**
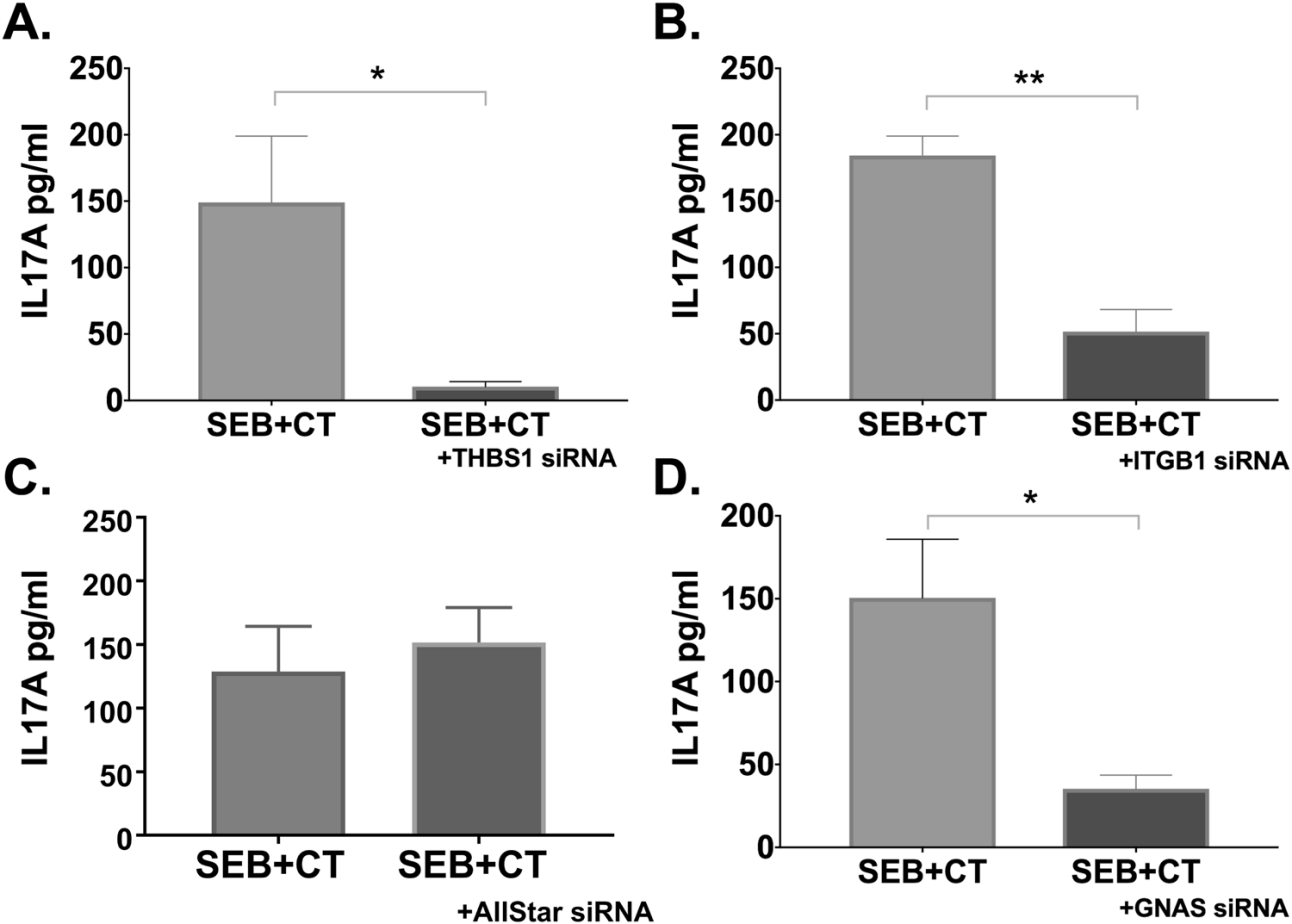
THSB1 and ITGB1-dependence of adjuvant effect of CT is also observed in human primary dendritic cells. Human primary dendritic cells purified from PBMCs were incubated with siRNAs specific for THSB1 (**A**), ITGB1 (**B**), All-Star Control (**C**), and GNAS (**D**) for 24 h. Cells were then left untreated or were treated for 16 h with CT, washed, and co-cultured with CD4^+^ T cells plus SEB for 3 days whereafter IL-17A levels in culture supernatants were measured by ELISA. Bars represent mean plus SEM of IL-17A levels.

To determine whether the dependence on THSB1 and ITGB1 for the adjuvant activity by CT found in human monocytes is also observed in another subset of APCs, experiments with purified DCs tested in co-culture with autologous CD4^+^ T cells were performed. Also similar to the studies with monocytes, inhibition of THSB1 or ITGB1 in DCs by specific or control siRNAs (Fig. 6A–D) followed by co-culture with T cells resulted in reduced Th17 responses. Thus, the THSB1 and ITGB1 dependent Th17-promoting adjuvant effect of CT found in monocytes was also found in DCs.

### Adjuvant effects of mmCT and dmLT on human monocytes also require THSB1 and ITGB1

**Figure 7:**
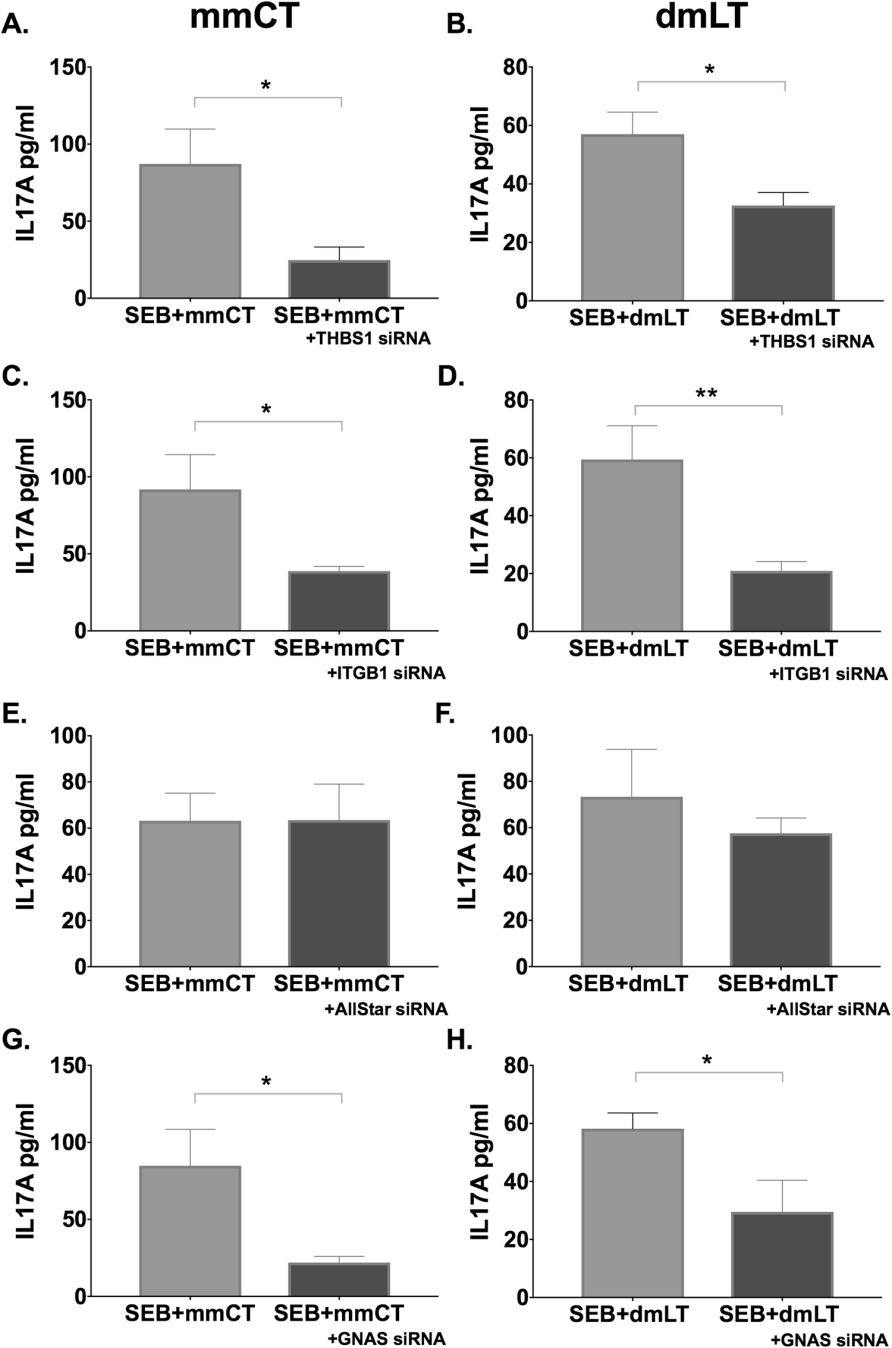
Adjuvant effect of mmCT and dmLT is also dependent on THBS1 and ITGB1. Purified CD14^+^ monocytes were incubated with siRNAs specific for THSB1 (**A**,**B**), ITGB1 (**C**,**D**), All-Star Control (**E**,**F**), and GNAS (**G**,**H**) for 24 h. Cells were then left untreated or were treated for 16 h with mmCT or dmLT, washed, and co-cultured for 3 days with autologous CD4^+^ T cells plus SEB. Bars represent mean plus SEM of IL-17A levels in culture supernatants measured by ELISA.

Lastly, we investigated whether THSB1 and ITGB1 play an important role also in the adjuvanticity of the almost non-toxic derivatives of CT or LT, mmCT and dmLT. It was previously shown that both mmCT and dmLT, despite their minimal induction of cAMP, promote Th17 responses via cAMP-PKA signaling^10^. Inhibition of THSB1 or ITGB1 with specific siRNAs in monocytes reduced the enhancement of Th17 responses by co-cultured T cells induced by mmCT or dmLT (Fig. 7A–F); the same was true for monocytes treated with siRNA specific for GNAS (Fig. 7G,H). Thus, the promotion of Th17 responses by CT, mmCT or dmLT requires at least a low level of cAMP response in adjuvant-treated APCs, and is, at least as tested here *in vitro* using human APCs, dependent on immuno-modulatory proteins THSB1 and ITGB1.

## Discussion

Mass spectrometry-based functional proteomics and recent advances in bioinformatics have provided new tools for identifying molecular mechanistic pathways of biological and pharmacologic agents including immune-enhancing adjuvants^24–27^. In this study we have undertaken high-throughput proteomic analysis of human monocytes after treatment with CT to gain new insights about immunomodulatory proteins and signaling pathways affected in APCs by this potent adjuvant. Previous work on the adjuvant function of CT and related molecules such as mmCT and dmLT has been done mainly in mice. However, these agents have strong anti-proliferative effects on murine APCs and lymphocytes^28^ which has seriously hampered *in vitro* studies of their adjuvant action. Human APCs and lymphocytes are not affected in the same way which is a major advantage when using these cells for more detailed studies of the molecular pathways involved in the adjuvant action of CT, mmCT and dmLT. Thus, in previous work using human immune cells *in vitro*, we and others have demonstrated that central to the adjuvant action of these agents is the activation of the cAMP/PKA pathway in APCs. Downstream processes including inflammasome-dependent IL1β production then lead to enhanced activation of helper T cells, predominantly Th17 but also Th1 and Th2 cells^7,10^. The strong promotion of Th17 cells is consistent with the potent mucosal adjuvanticity of CT, mmCT and dmLT, since several studies have identified IL-17 as a cytokine of special importance for enhancing secretory IgA responses at mucosal surfaces^29–31^.

In the present study we show, for the first time, that CT strongly upregulates the expression of many proteins, in most cases peaking at 16 rather than at 2–6 hours post-stimulation, that can be grouped in three main functional categories: cellular organization, metabolism, and immune response. The orchestrated induction of these sets of proteins by CT appears to characterize the shift from a steady-state “resting” monocyte into a dynamic, energy-demanding and immunologically active APC, and is consistent with similar protein expression dynamics induced by other adjuvants such as alum, LPS, and TLR receptor agonists^32,33^.

The “structural reorganization” proteins induced by CT, which include proteins promoting cell motility and extracellular matrix (ECM) interactions, is an early step in the activation of APCs. When immature dendritic cells or monocytes take up antigen together with a co-administered adjuvant at local sites of vaccine administration, they become activated and turn into effective APCs. This requires structural changes that enable the cells to migrate to local lymph nodes to facilitate APC interaction with cognate T cells, and hence initiate an adaptive immune response. Such migration of APCs from a local exposure site to a nearby lymph node is a complex process, involving the interplay of various integrins and related proteins including several proteins we found to be increased in monocytes after CT exposure: CD44, ITGB1, THSB1, COP, Syntenin-1, and SCLA3A2^32,34–39^.

APC activation is also a metabolically demanding process, and requires efficient production and utilization of energy. Commonly used adjuvants have been found to induce a metabolic shift in APCs including activation of carbohydrate metabolism^40^ and/or increased expression of mitochondrial proteins^41^. In CT-exposed monocytes, we observed increased expression of subunits of NADH:ubiquinone oxidoreductase, NDUFA9 and NDUFB9, which all play a role in complex I activity of the electron transport chain located in the inner mitochondrial membrane^42^. This is consistent with previous findings demonstrating cAMP/PKA-dependent induction by CT or dcAMP of energy production regulating mitochondrial complex I activity in murine fibroblasts^43^.

In this study, we show that two immune-related proteins, THSB1 and ITGB1, are essential in the adjuvant action of CT on human APCs *in vitro*. Both proteins were significantly overexpressed in response to CT treatment, and inhibition of either of these proteins resulted in abrogation of the adjuvant effect. THSB1 is a glycoprotein known to be involved in cellular organization necessary for chemotaxis and haptotaxis^44–46^. It binds to wide array of substances, including protein and non-protein glycans, ranging from extracellular matrix molecules, cell surface receptors, growth regulatory factors and protease enzymes^47^. Interestingly, the N-terminal pentraxin module of THSB1 was shown to be a ligand for ITGB1^48,49^, with binding of THSB1 to ITGB1 activating various biological processes such as chemotaxis, cellular proliferation, and angiogenesis^50–52^. ITGB1 in its turn is a membrane protein belonging to the integrin subfamily, which is comprised mainly of the receptors for ECM proteins^53^. It has been previously shown that the ligand/ITGB1 interaction results in strong activation via the NF-KB transcription factor of inflammatory mediators, such as IL-1, IL-8 and Tumour necrosis factor alpha (TNFα) and early genes characteristic of monocytic activation^47–49,52,54^. Also, aside from promoting IL-1 proform production, ITGB1-binding domains of THSB1 independently enhance the inflammasome-dependent maturation of IL-1β in human THP-1 monocyte-derived macrophages^55^. In our system, THSB1 interacting with the ITGB1 may provide an autocrine or paracrine loop that is important in the monocyte activation by CT leading to enhanced NFKB/inflammasome-dependent IL-1β production and maturation.

The adjuvant effect of CT is mainly cAMP/PKA/NFKB-dependent. Even a small amount of cAMP is enough to induce NFKB-dependent IL-1β production, inasmuch as mmCT and dmLT even though being 1000-fold less potent than CT in inducing cAMP can provide similar Th17 promotion via IL-1β release by APCs as achieved with CT. We show here that cAMP/PKA via NFKB activation is also crucial for CT-, mmCT-, or dmLT-induced THSB1 and ITG1 expression. Furthermore, and of particular functional importance, we show that the inhibition of either of these proteins by specific siRNAs strongly abrogated the adjuvant action of CT, mmCT or dmLT in both monocytes and DCs, as did also the inhibition of PKA or GNAS in the APCs.

Interestingly, while the present study points to a strong unidirectional immunostimulatory role of THSB1 and ITGB1 in APCs in response to CT, mmCT or dmLT, several studies in THSB1^−/−^ mice have suggested that THSB1 can exert both pro- and anti-inflammatory effects^56^, with the latter proposed to serve as an immunoregulatory brake preventing excessive tissue damage after immune stimulation. It remains to be defined to which extent the immunostimulatory effects of THSB1 and ITGB1 found in human APCs after treatment with CT and related adjuvants *in vitro* would be manifested also *in vivo*. It could be that in THSB1^−/−^ mice, existing compensatory effects may mask a specialized immunostimulatory function of THSB1 in APCs; a way to address this issue in future studies could be to use Cre/*lox* APC-specific inducible knockout mice. Alternatively, the reported binding properties of THSB1 to multiple receptors^47^, possibly in a concentration-dependent manner, might differentially modulate the balance in favor of immune enhancement or immune regulation.

Altogether, we show that although mass spectrometry-based proteomics is associated with significant challenges^25^, including limited ability to detect low abundance proteins such as inflammatory cytokines^57^, it was able to identify proteins that were previously not appreciated as engaged in the adjuvant effect of CT and related nontoxic derivatives. Our results suggest that as tested on human APCs and T cells *in vitro* the adjuvant activity of CT, mmCT and dmLT is regulated by THSB1 and ITGB1, and that the adjuvant-induced expression of these proteins in human APCs similar to other observed adjuvant mechanisms by these agents is cAMP/PKA/NFKB-dependent. These findings provide valuable novel insights in the mechanism of action of cAMP-inducing adjuvants, and may aid in the rational design of future vaccines.

## Supplementary information

Supplemental file 1

Supplementary_Table_1

Supplementary_Table_2

Supplementary_Table_3

Supplementary_Table_4

## References

[1] Lycke N, Holmgren J (1988). Mucosal immune response to cholera toxin–cellular basis of memory and adjuvant action. Monogr. Allergy.

[2] Chong C, Friberg M, Clements JD (1998). LT(R192G), a non-toxic mutant of the heat-labile enterotoxin of Escherichia coli, elicits enhanced humoral and cellular immune responses associated with protection against lethal oral challenge with Salmonella spp. Vaccine.

[3] Lycke N, Bemark M (2010). Mucosal adjuvants and long-term memory development with special focus on CTA1-DD and other ADP-ribosylating toxins. Mucosal Immunol..

[4] Norton EB, Lawson LB, Freytag LC, Clements JD (2011). Characterization of a mutant Escherichia coli heat-labile toxin, LT(R192G/L211A), as a safe and effective oral adjuvant. Clin. Vaccine Immunol..

[5] Lebens M (2016). Construction and preclinical evaluation of mmCT, a novel mutant cholera toxin adjuvant that can be efficiently produced in genetically manipulated Vibrio cholerae. Vaccine.

[6] Clemens JD, Nair GB, Ahmed T, Qadri F, Holmgren J (2017). Cholera. Lancet.

[7] Gagliardi, M. C. *et al*. Cholera toxin induces maturation of human dendritic cells and licences them for Th2 priming. *Eur. J. Immunol*. **30**, 2394–2403, doi:10.1002/1521-4141(2000)30:8<2394::AID-IMMU2394>3.0.CO;2-Y (2000). 10.1002/1521-4141(2000)30:8<2394::AID-IMMU2394>3.0.CO;2-Y 10940931

[8] Lavelle EC (2003). Cholera toxin promotes the induction of regulatory T cells specific for bystander antigens by modulating dendritic cell activation. J. Immunol..

[9] Veglia F (2011). Cholera toxin impairs the differentiation of monocytes into dendritic cells, inducing professional antigen-presenting myeloid cells. Infect. Immun..

[10] Larena M, Holmgren J, Lebens M, Terrinoni M, Lundgren A (2015). Cholera toxin, and the related nontoxic adjuvants mmCT and dmLT, promote human Th17 responses via cyclic AMP-protein kinase A and inflammasome-dependent IL-1 signaling. J. Immunol..

[11] Sun JB (2017). Deficiency in Calcium-Binding Protein S100A4 Impairs the Adjuvant Action of Cholera Toxin. Front. Immunol..

[12] Mattsson J (2015). Cholera toxin adjuvant promotes a balanced Th1/Th2/Th17 response independently of IL-12 and IL-17 by acting on Gsalpha in CD11b(+) DCs. Mucosal Immunol..

[13] Riccomi A, Gesa V, Sacchi A, De Magistris MT, Vendetti S (2016). Modulation of Phenotype and Function of Human CD4(+) CD25(+) T Regulatory Lymphocytes Mediated by cAMP-Elevating Agents. Front. Immunol..

[14] Batth TS, Francavilla C, Olsen JV (2014). Off-line high-pH reversed-phase fractionation for in-depth phosphoproteomics. J. Proteome Res..

[15] Yang F, Shen Y, Camp DG, Smith RD (2012). High-pH reversed-phase chromatography with fraction concatenation for 2D proteomic analysis. Expert Rev Proteomics.

[16] Vizcaino JA (2013). The PRoteomics IDEntifications (PRIDE) database and associated tools: status in 2013. Nucleic Acids Res..

[17] Liu P, Beer LA, Ky B, Barnhart KT, Speicher DW (2017). Quantitative Comparisons of Large Numbers of Human Plasma Samples Using TMT10plex Labeling. Methods Mol. Biol..

[18] Mann M, Kelleher NL (2008). Precision proteomics: the case for high resolution and high mass accuracy. Proc. Natl. Acad. Sci. USA.

[19] Serang O, Cansizoglu AE, Kall L, Steen H, Steen JA (2013). Nonparametric Bayesian evaluation of differential protein quantification. J. Proteome Res..

[20] Huang da W, Sherman BT, Lempicki RA (2009). Bioinformatics enrichment tools: paths toward the comprehensive functional analysis of large gene lists. Nucleic Acids Res..

[21] Huang da W, Sherman BT, Lempicki RA (2009). Systematic and integrative analysis of large gene lists using DAVID bioinformatics resources. Nat. Protoc..

[22] Mi H, Muruganujan A, Casagrande JT, Thomas PD (2013). Large-scale gene function analysis with the PANTHER classification system. Nat. Protoc..

[23] Pearce EJ, Everts B (2015). Dendritic cell metabolism. Nat. Rev. Immunol..

[24] Thompson A (2003). Tandem mass tags: a novel quantification strategy for comparative analysis of complex protein mixtures by MS/MS. Anal. Chem..

[25] Kocher T, Superti-Furga G (2007). Mass spectrometry-based functional proteomics: from molecular machines to protein networks. Nat. Methods.

[26] Oh DY (2016). Adjuvant-induced Human Monocyte Secretome Profiles Reveal Adjuvant- and Age-specific Protein Signatures. Mol. Cell. Proteomics.

[27] Nohr MK (2016). SILAC-MS Based Characterization of LPS and Resveratrol Induced Changes in Adipocyte Proteomics - Resveratrol as Ameliorating Factor on LPS Induced Changes. PLoS One.

[28] Holmgren J, Lindholm L, Lonnroth I (1974). Interaction of cholera toxin and toxin derivatives with lymphocytes. I. Binding properties and interference with lectin-induced cellular stimulation. J. Exp. Med..

[29] Datta SK (2010). Mucosal adjuvant activity of cholera toxin requires Th17 cells and protects against inhalation anthrax. Proc. Natl. Acad. Sci. USA.

[30] Hirota K (2013). Plasticity of Th17 cells in Peyer’s patches is responsible for the induction of T cell-dependent IgA responses. Nat. Immunol..

[31] Leach S, Clements JD, Kaim J, Lundgren A (2012). The adjuvant double mutant Escherichia coli heat labile toxin enhances IL-17A production in human T cells specific for bacterial vaccine antigens. PLoS One.

[32] Mosca F (2008). Molecular and cellular signatures of human vaccine adjuvants. Proc. Natl. Acad. Sci. USA.

[33] Olafsdottir T, Lindqvist M, Harandi AM (2015). Molecular signatures of vaccine adjuvants. Vaccine.

[34] Chen H, Herndon ME, Lawler J (2000). The cell biology of thrombospondin-1. Matrix Biol..

[35] Deves R, Boyd CA (2000). Surface antigen CD98(4F2): not a single membrane protein, but a family of proteins with multiple functions. J. Membr. Biol..

[36] Mitchell DM, Williams MA (2010). An activation marker finds a function. Immunity.

[37] Heinrich C (2010). Copine-III interacts with ErbB2 and promotes tumor cell migration. Oncogene.

[38] Sala-Valdes M (2012). Association of syntenin-1 with M-RIP polarizes Rac-1 activation during chemotaxis and immune interactions. J. Cell Sci..

[39] Sjokvist Ottsjo L (2017). Induction of mucosal immune responses against Helicobacter pylori infection after sublingual and intragastric route of immunization. Immunology.

[40] Everts B, Pearce EJ (2014). Metabolic control of dendritic cell activation and function: recent advances and clinical implications. Front. Immunol..

[41] Krawczyk CM (2010). Toll-like receptor-induced changes in glycolytic metabolism regulate dendritic cell activation. Blood.

[42] Papa S (2012). Respiratory chain complex I, a main regulatory target of the cAMP/PKA pathway is defective in different human diseases. FEBS Lett..

[43] Scacco S (2000). cAMP-dependent phosphorylation of the nuclear encoded 18-kDa (IP) subunit of respiratory complex I and activation of the complex in serum-starved mouse fibroblast cultures. J. Biol. Chem..

[44] Mansfield PJ, Suchard SJ (1994). Thrombospondin promotes chemotaxis and haptotaxis of human peripheral blood monocytes. J. Immunol..

[45] Reyes-Reyes M, Mora N, Zentella A, Rosales C (2001). Phosphatidylinositol 3-kinase mediates integrin-dependent NF-kappaB and MAPK activation through separate signaling pathways. J. Cell Sci..

[46] Reyes-Reyes M, Mora N, Gonzalez G, Rosales C (2002). beta1 and beta2 integrins activate different signalling pathways in monocytes. Biochem. J..

[47] Resovi A, Pinessi D, Chiorino G, Taraboletti G (2014). Current understanding of the thrombospondin-1 interactome. Matrix Biol..

[48] Calzada MJ (2004). Identification of novel beta1 integrin binding sites in the type 1 and type 2 repeats of thrombospondin-1. J. Biol. Chem..

[49] Calzada MJ (2004). Alpha4beta1 integrin mediates selective endothelial cell responses to thrombospondins 1 and 2 *in vitro* and modulates angiogenesis *in vivo*. Circ. Res..

[50] Chandrasekaran S, Guo NH, Rodrigues RG, Kaiser J, Roberts DD (1999). Pro-adhesive and chemotactic activities of thrombospondin-1 for breast carcinoma cells are mediated by alpha3beta1 integrin and regulated by insulin-like growth factor-1 and CD98. J. Biol. Chem..

[51] Chandrasekaran L (2000). Cell contact-dependent activation of alpha3beta1 integrin modulates endothelial cell responses to thrombospondin-1. Mol. Biol. Cell.

[52] Klein S (2002). Alpha 5 beta 1 integrin activates an NF-kappa B-dependent program of gene expression important for angiogenesis and inflammation. Mol. Cell. Biol..

[53] Giancotti FG, Ruoslahti E (1999). Integrin signaling. Science.

[54] Xing T (2017). Thrombospondin-1 Production Regulates the Inflammatory Cytokine Secretion in THP-1 Cells Through NF-kappaB Signaling Pathway. Inflammation.

[55] Stein EV, Miller TW, Ivins-O’Keefe K, Kaur S, Roberts DD (2016). Secreted Thrombospondin-1 Regulates Macrophage Interleukin-1beta Production and Activation through CD47. Sci. Rep..

[56] Lopez-Dee Z, Pidcock K, Gutierrez LS (2011). Thrombospondin-1: multiple paths to inflammation. Mediators Inflamm..

[57] Stenken JA, Poschenrieder AJ (2015). Bioanalytical chemistry of cytokines–a review. Anal. Chim. Acta.

