# Supplementary material for: Proteomic analysis of cholera toxin adjuvant-stimulated human monocytes identifies Thrombospondin-1 and Integrin-β1 as strongly upregulated molecules involved in adjuvant activity
Source: Sci Rep. 2019 Feb 26;9:2812. doi: 10.1038/s41598-019-38726-0 (PMC6391456; doi:10.1038/s41598-019-38726-0)
Supplement: Supplementary file 1 — Supplemental file 1 [file 41598_2019_38726_MOESM1_ESM.pdf]

SUPPLEMENTAL FILE 1

Proteomic analysis of cholera toxin adjuvant-stimulated human monocytes identifies Thrombospondin-1 and Integrin-β1 as strongly upregulated molecules involved in adjuvant activity.

Manuela Terrinoni[1], *Jan Holmgren*[1], *Michael Lebens*[1], *Maximilian Larena*[1, 2]*

Authors affiliations:

[1]Department of Microbiology and Immunology and University of Gothenburg Vaccine Research Institute (GUVAX), Institute of Biomedicine, Sahlgrenska Academy at University of Gothenburg, Box 435, SE-405 30 Gothenburg, Sweden.

[2] Department of Organismal Biology, Uppsala University, Norbyvägen 18C, SE-753 26, Uppsala, Sweden

**Corresponding author:* Maximilian Larena, Department of Microbiology and Immunology, Institute of Biomedicine, Sahlgrenska Academy, University of Gothenburg, Medicinaregatan 7A, 41190 Gothenburg, Sweden. Email: maximilian.larena@gu.se

Telephone: +46 184712624

Running title: proteomic studies, adjuvant action, mucosal adjuvants, Cholera toxin, mmCT, dmLT

Exp 1		Exp 2	
**SAMPLE ID**	**TMT label**	**SAMPLE ID**	**TMT label**
**NS-1**	**126TM**	**$CT_{time\ 4h}$-1**	**126TM**
**NS-2**	**127N**	**$CT_{time\ 4h}$-2**	**127N**
**NS-3**	**127C**	**$CT_{time\ 4h}$-3**	**127C**
**$CT_{time\ 2h}$-1**	**128N**	**$CT_{time\ 6h}$-1**	**128N**
**$CT_{time\ 2h}$-2**	**128C**	**$CT_{time\ 6h}$-2**	**128C**
**$CT_{time\ 2h}$-3**	**129N**	**$CT_{time\ 6h}$-3**	**129N**
**X-1**	**129C**	**$CT_{time\ 16h}$-1**	**129C**
**X-2**	**130N**	**$CT_{time\ 16h}$-2**	**130N**
**X-3**	**130C**	**$CT_{time\ 16h}$-3**	**130C**
**Pool**	**131TM**	**Pool**	**131TM**

Table S1. TMT 10plex labeling. (X: treatment outside the current study). As explained in Methods, each sample represents the digested pooled proteins from 6 individuals.

Time (min)	%B
0	3
5	3
125	25
135	60
140	70
145	70
150	3
160	3

Table S2. HpH RP (High pH Reversed-Phase) Gradient.

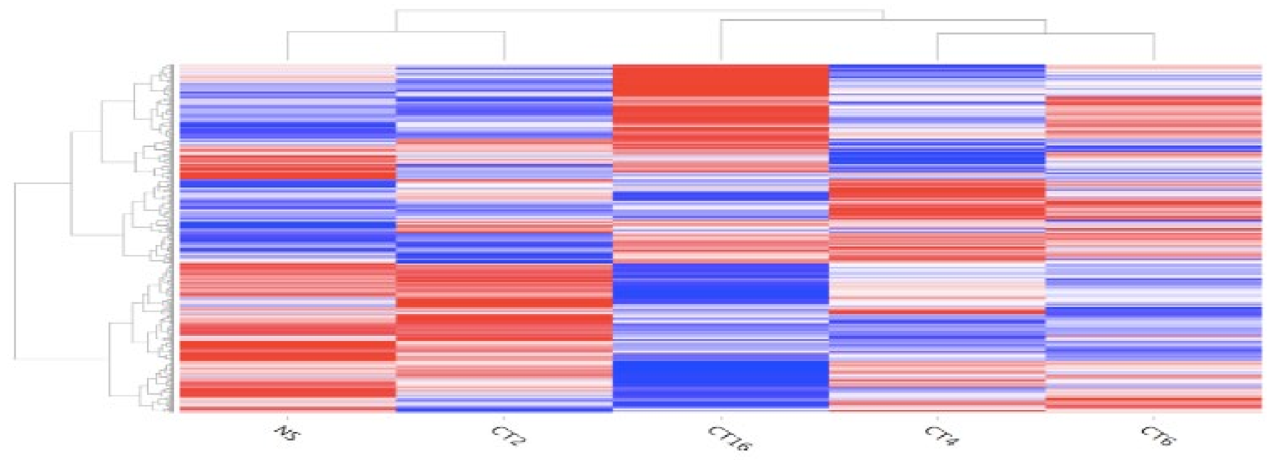

Figure S1. Hierarchical Cluster analysis of CT stimulated monocytes indicates differences in protein distribution over time. The blue-to-red color scale represents the gene expression values going from low to high.

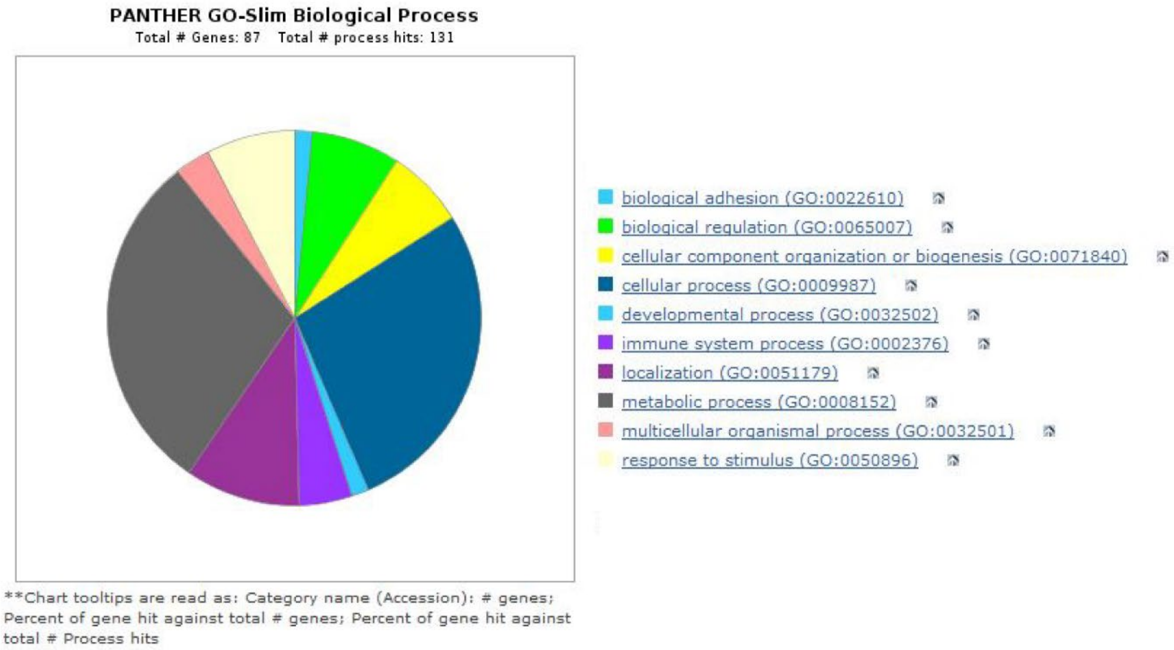

Figure S2. Panther software diagram showing the main biological processes in CT-treated human monocytes at 16h.

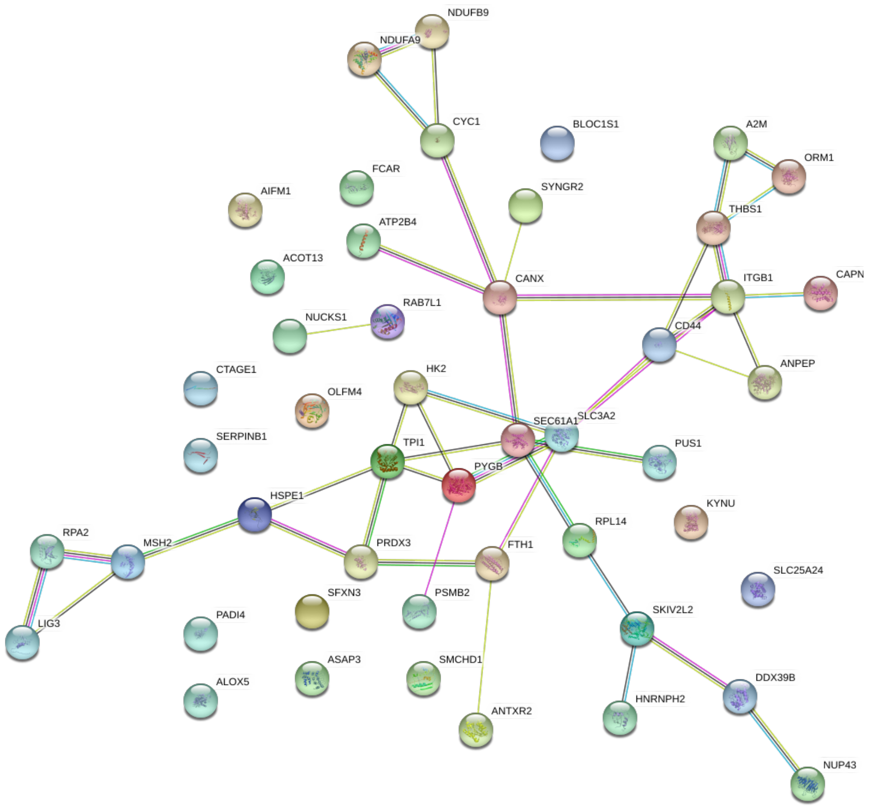

Figure S3. STRING software diagram of networks of upregulated proteins in CT-treated monocytes at 16h.

**Treatments**	**Live CD14$^+$ Cell x counts**	**% Trypan blue positive**
**NS** $_{\textbf{time 0}}$	1.02±0.028 x 10^6/ml	**<1%**
**NS** $_{\textbf{time 2h}}$	0.99±0.052 x10^6/ml	**< 1%**
**NS** $_{\textbf{time 16h}}$	1.00±0.013 x 10^6/ml	**< 1%**
**CT** $_{\textbf{time 16h}}$	1.02±0.038 x 10^6/ml	**< 1%**
**CT+H-89** $_{\textbf{time 16h}}$	1.03±0.026 x 10^6/ml	**< 1%**
**CT+CAPE** $_{\textbf{time 16h}}$	1.00±0.041x 10^6/ml	**< 1%**

Table S3. Live CD14$^+$ cell counts and % Trypan blue positive cells (dead cells) at different times of incubation of non-stimulated (NS) CD14$^+$ human monocytes and similar cells treated with cholera toxin (CT), CT + protein kinase A inhibitor H-89, or CT + NFKB inhibitor CAPE as described in Methods.
